# DARG: An integrated knowledge base for analyzing addictive drug-related genes

**DOI:** 10.1016/j.gendis.2024.101369

**Published:** 2024-06-28

**Authors:** Xu Wang, Bei Yun, Zihan Zhang, Xiaoxi Wang, Yifan Wu, Yubo Hu, Shiyi Fang, Junjie Lv, Lina Chen, Wan Li

**Affiliations:** College of Bioinformatics Science and Technology, Harbin Medical University, Harbin, Heilongjiang 150081, China

Drug addiction is a complex brain disease closely related to the expression and methylation of many genes. Differential genes can influence addiction, and some key differential genes may have a significant impact on the occurrence and development of addiction. Nowadays, data on addictive drugs is widely available. To our knowledge, there are very few databases on genes related to addictive drugs, and existing addiction related databases are not available.^1^^,^^2^ Therefore, it is necessary to analyze existing data on drug addiction, identify genes that play a crucial role in addiction, and establish a database to store and display these results. To meet this requirement, we analyzed and integrated the results of 56 datasets from 10 addictive drugs to develop DARG (Drug Addiction Related Gene Database) (https://darg-database.cn/). Through various search modules, corresponding analysis results can be obtained, including differential analysis results, protein–protein interaction (PPI) network analysis results, centrality analysis results, enrichment analysis results, gene–drug correlation analysis results, *etc*. Meanwhile, DARG provides online analysis functionality, allowing users to upload a gene list to obtain key genes within these genes. DARG will be a valuable resource for studying addiction related genes, providing great convenience for researchers and clinical doctors.

We conducted a comprehensive search on addictive drug related data in the GEO database using addictive drugs as the keyword. A total of 47 transcriptome datasets (including 31 sequencing datasets and 16 chip datasets) for 10 addictive drugs and 9 methylation datasets for 5 addictive drugs were obtained (Supplementary Data 1). The transcriptome dataset was derived from 27 different brain regions, and the methylation dataset originated from 6 different tissues/cells.

For the downloaded sequencing and chip data, we used the DESeq2 and limma packages for differential expression analysis, respectively. Differential expression genes (DEGs) were screened using multiple thresholds. The CHAMP software package 3 was used to analyze methylation data. Differential methylation probes and corresponding genes were screened using multiple thresholds. The ProbeLasso method was used to identify differentially methylated regions and corresponding genes, with a threshold of *P* < 0.05.

The PPI data for humans and mice were downloaded from the STRING database (https://string-db.org/, v12.0), with a comprehensive score >700 as the threshold. A differentially expressed gene interaction network was constructed with gene interactions as the edges and DEGs as the nodes, where the gene interaction relationships were obtained by filtering and downloading PPI data using different DEGs. We proposed a centrality algorithm integration strategy to analyze genes in differential expression gene interaction networks. The scores of the node in networks under each centrality algorithm were calculated separately by applying a series of centrality measures, including degree, edge-percolated component, Laplacian centrality, maximum neighborhood component, Katz radiality, and semi-local centrality. The intersection of the top 10% genes of each centrality algorithm was considered the key genes.^3^

To investigate the possible molecular mechanisms of DEGs, functional enrichment and pathway analysis were conducted using Gene Ontology (GO) and Kyoto Encyclopedia of Genes and Genomes (KEGG) databases. The genes in the differential expression gene interaction networks were enriched and analyzed using the clusterProfiler software package in R software, while the differential methylation probes were enriched and analyzed using the methylGSA software package^4^ in R software.

All analysis results were integrated to construct DARG, which has a user-friendly web interface, allowing users to query the database (Fig. 1) through several flexible steps. Users can click on the buttons on the navigation bar to enter the “Home”, “Search”, “Analyse”, “Help”, and “Download” pages.

**Figure 1:**
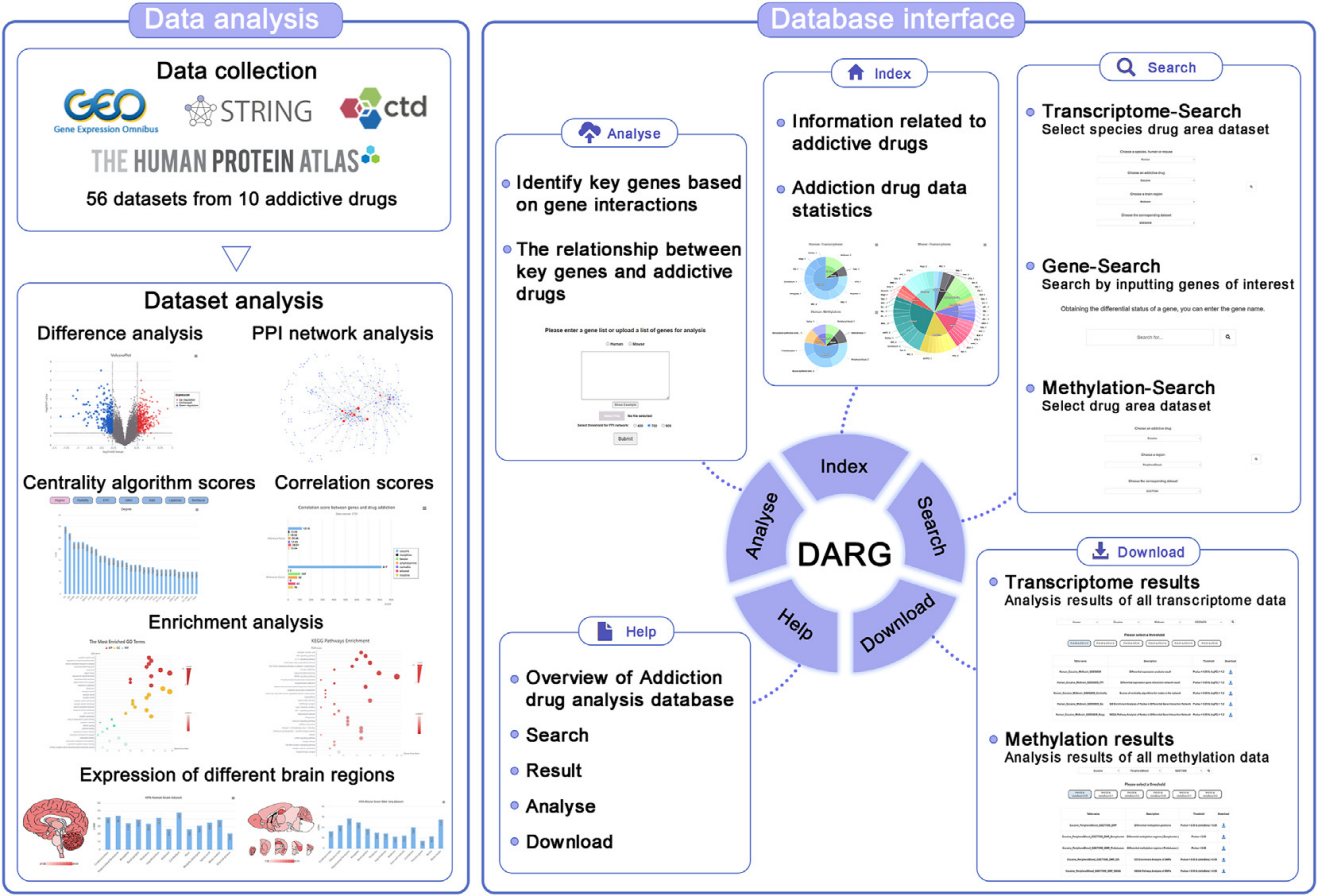
The content and interface of DARG. The “Data analysis” displays the analysis and processing process of all data, and the “Database interface” displays the user interface of DARG.

The “Home” page displays an introduction to DARG and relevant information about the data it contains. On the “Search” module, DARG provides three search methods, namely transcriptome data search, gene search, and methylation data search. On the “Transcriptome Data Search” page, users can select the species, drugs, brain regions, and datasets of interest to search results for transcriptome data. On the “Gene Search” page, users can input the gene of interest to obtain the differential expression and methylation results of that gene in all datasets. On the “Methylation Data Search” page, users can select the drugs, tissues/cells, and datasets of interest to search for the analysis results of methylation data. On the “Analyse” page, users can upload a gene list and select a threshold to obtain the corresponding analysis results. The “Help” page provides a detailed explanation of how to use DARG. On the “Download” page, users can download all analysis results by selecting the transcriptome dataset or methylation dataset of interest and different thresholds (See Supplementary Data 2 for details).

DARG was written using Django (a Python network framework) with all data stored in the MySQL (v 8.0.17) database. The images on the page were generated by Highcharts 7.1.2 and Echarts 5.2.2. The website has been tested on several popular web browsers, including Google Chrome, Firefox, and Microsoft Edge browsers.

DARG provides users with online analysis functionality. Firstly, it constructs a PPI network based on the gene list uploaded by the user and selected thresholds. Subsequently, employing our proposed centrality algorithm integration strategy, it calculates scores for network nodes using seven different centrality algorithms. The intersection of the top 10% of genes from each centrality algorithm is regarded as the set of key genes. Additionally, DARG furnishes users with information on the relationship between these key genes and addictive drugs.

In summary, DARG provides analysis results of transcriptome and methylation data for multiple addictive drugs, making it an important resource for studying addictive drugs. User-friendly interface was designed for querying, browsing, analyzing, and downloading relevant analysis results. In addition, there is currently an increasing amount of single-cell data, and in the future, we will add single-cell related results to the database to provide more analysis results of addiction drug data. We believe that DARG provides great convenience for researchers and clinical doctors, and is a valuable resource for studying addictive drugs.

## Author contributions

Conceptualization, W.L. and L.C.; data curation, X.W., B.Y., Z.Z., X.X.W., Y.W., Y.H., S.F., and J.L.; formal analysis, X.W.; funding acquisition, W.L.; investigation, X.W.; methodology, X.W., W.L. and L.C.; project administration, W.L. and L.C.; supervision, W.L. and L.C.; validation, B.Y., Z.Z., X.X.W., Y.W., Y.H., S.F., and J.L.; visualization, X.W. and W.L.; writing—original draft, X.W. and W.L.; writing—review and editing, L.C. All authors read and agreed to the published version of the manuscript.

## Funding

This work was supported by the 10.13039/501100005046Natural Science Foundation of Heilongjiang Province, China (No. LH2021F043) and the National Natural Science Foundation of China (No. 61702141).

## Data availability

Publicly available datasets were analyzed in this study. These data can be found at https://www.ncbi.nlm.nih.gov/geo/.

## Conflict of interests

The authors declared that the research was conducted in the absence of any commercial or financial relationships that could be construed as a potential conflict of interest.
