# Supplementary material for: DARG: An integrated knowledge base for analyzing addictive drug-related genes
Source: Genes Dis. 2024 Jun 28;12(2):101369. doi: 10.1016/j.gendis.2024.101369 (PMC11635640; doi:10.1016/j.gendis.2024.101369)
Supplement: Multimedia component 1 [file mmc1.docx]

**Supplementary Data**

**Results:**

**Database content:**

DARG includes the analysis results of transcriptome data and methylation data. All transcriptome data were subjected to differential expression analysis, gene interaction network analysis, GO and KEGG enrichment analysis. Utilizing seven centrality algorithms, we computed centrality scores for genes within the differential expression gene interaction networks and identified key genes based on intersections. For the methylation data, we analysed DMPs and DMRs, and performed GO and KEGG enrichment analysis based on DMPs with varying thresholds.

**Search Interface:**

In the search module, we provided three search methods: Transcriptome data Search, Gene Search, and Methylation data Search. On the "Transcriptome data Search" page, users can select species, drug, brain region, and dataset of interest to search for transcriptome data analysis results. For example, by selecting "human", "cocaine", "midbrain", and "GSE54839" (Fig S1A), and then clicking the search button, users can obtain detailed analysis results for the dataset (The default threshold for differentially expressed genes is p<0.05 and | FoldChange |>1.2) (Figs S1B, C, D, E). In the PPI network, the intersection of the top 10% of genes for each centrality algorithm is considered the key gene. In the network diagram, red color represents key genes. The analysis results of different thresholds can be viewed by clicking on the numbers in the DEGs table. Users can click on the GO and KEGG entries to jump to a detailed introduction of functions and pathways. In addition, users can click the icon next to the number to view a detailed list of DEGs corresponding to the threshold (Fig S2A). Subsequently, by clicking on the gene name, the user can jump to the gene details page (Figs S2B, C, D). The expression levels of genes in different brain regions were obtained from the Human Protein Atlas (HPA; https://www.proteinatlas.org/) database[1]. The correlation between genes and addictive drugs and the literature information about genes and addictive drugs were obtained from the Comparative Toxicogenomics Database (CTD, <https://ctdbase.org/>). In the CTD, the inference score reflects the degree of similarity between the CTD chemical-gene-disease network and a similar scale-free random network [2], and the reference count is the number of literature related to the association between the gene and the disease. The higher the inference score and reference count, the higher the degree of association between the disease and the gene.

On the "Gene Search" page, users can enter a gene of interest, such as "ADCK4"(Fig S4A), and then click the search button to obtain the differential expression and methylation results of the gene in all datasets (Fig S3B). At the top of the page are the differential methylation probes results. At the bottom of the page are the differential expression results. Users can click on the gene name to obtain detailed information about the gene.

On the "Methylation data Search" page, users can select the drug, tissue/cell, and dataset of interest to search for the analysis results of methylation data. For example, by selecting "Nicotine," "DLPFC," and "GSE90871" (Fig S4A), then click the search button to obtain the dataset analysis results (Figs S4B, C, D). The analysis results of different thresholds can be viewed by clicking on the numbers in the DMPs table. In addition, users can click on the icon next to the number in the DMPs table or the number in the DMRs table to navigate to the respective detailed list pages. (Fig S5A, B). On the detailed list page of DMPs or DMRs, users can click on the relevant gene name of the DMPs or DMRs to view results related to the addictive drugs (Fig S5C, D).

**Analyse Interface:**

DARG provides users with online functionality for constructing a PPI network based on uploaded genes and selected thresholds to identify key genes. (Fig S6A). On the "Analyse" page, users could enter a gene list or click on the "Show Examples" button, then click "Search" to obtain the interaction network of these genes, results from centrality algorithms, and key genes selected based on centrality algorithms (Figs S6B, C). Click on a gene name to access information about the relationship between the key gene and addictive drugs (Figs S7A, B, C, D).

**Download Interface:**

On the "Download" page, users can download all analysis results by selecting the transcriptome dataset or methylation dataset of interest and different thresholds (Figs S8A, B).

**Future plans**

We will continuously update and improve DARG in the coming years to include more data on addictive drugs. The data on addictive drugs will be added to the project through annual searches from the GEO database. When the public database we used is updated, their related data will be updated.

**References：**

1. Sjöstedt, E., et al., *An atlas of the protein-coding genes in the human, pig, and mouse brain.* Science, 2020. **367**(6482).

2. Davis, A.P., et al., *Comparative Toxicogenomics Database (CTD): update 2023.* Nucleic Acids Res, 2023. **51**(D1): p. D1257-d1262.

**Supplementary figure legends**

**Figure S1** Transcriptome data Search page and analyse results: (A) Transcriptome data Search page. (B) DEGs table with different thresholds and volcano plots of DEGs. (C) Differential gene interaction network and centrality algorithm results. (D) GO enrichment result and KEGG pathway result.

**Figure S2** DEGs list and detailed information: (A) Detailed list of DEGs. (B) Box plot of gene expression levels and homologous gene information. (C) Correlation between gene and addictive drugs and literature information on gene and addictive drugs. (D) RNA expression in human and mouse brain regions.

**Figure S3** Gene Search page and result: (A) Gene Search page. (B) Search results for differential expression and differential methylation.

**Figure S4** Methylation data Search page and detailed information: (A) Methylation data Search page. (B) DMPs table with different thresholds and DMRs table. (C) Volcano map of DMPs. (D) GO enrichment result and KEGG pathway result.

**Figure S5** DMPs List and Gene Details: (A) Detailed list of DMPs and related genes. (B) Detailed list of DMRs and related genes. (C) Detailed analysis results of genes corresponding to DMPs. (D) Detailed analysis results of genes corresponding to DMRs.

**Figure S6** Analyse upload and result page: (A) Analyse upload page. (B) Gene interaction network. (C) Centrality algorithm results and key gene list.

**Figure S7** Key gene page in the analysis results: (A) Homologous gene information. (B) Correlation between gene and addictive drugs and literature information on gene and addictive drugs. (C) RNA expression in human brain regions. (D) RNA expression in mouse brain regions.

**Figure S8** Download page: (A) Transcriptome data analyse results download page. (B) Methylation data analyse results download page.

**Supplementary figures**


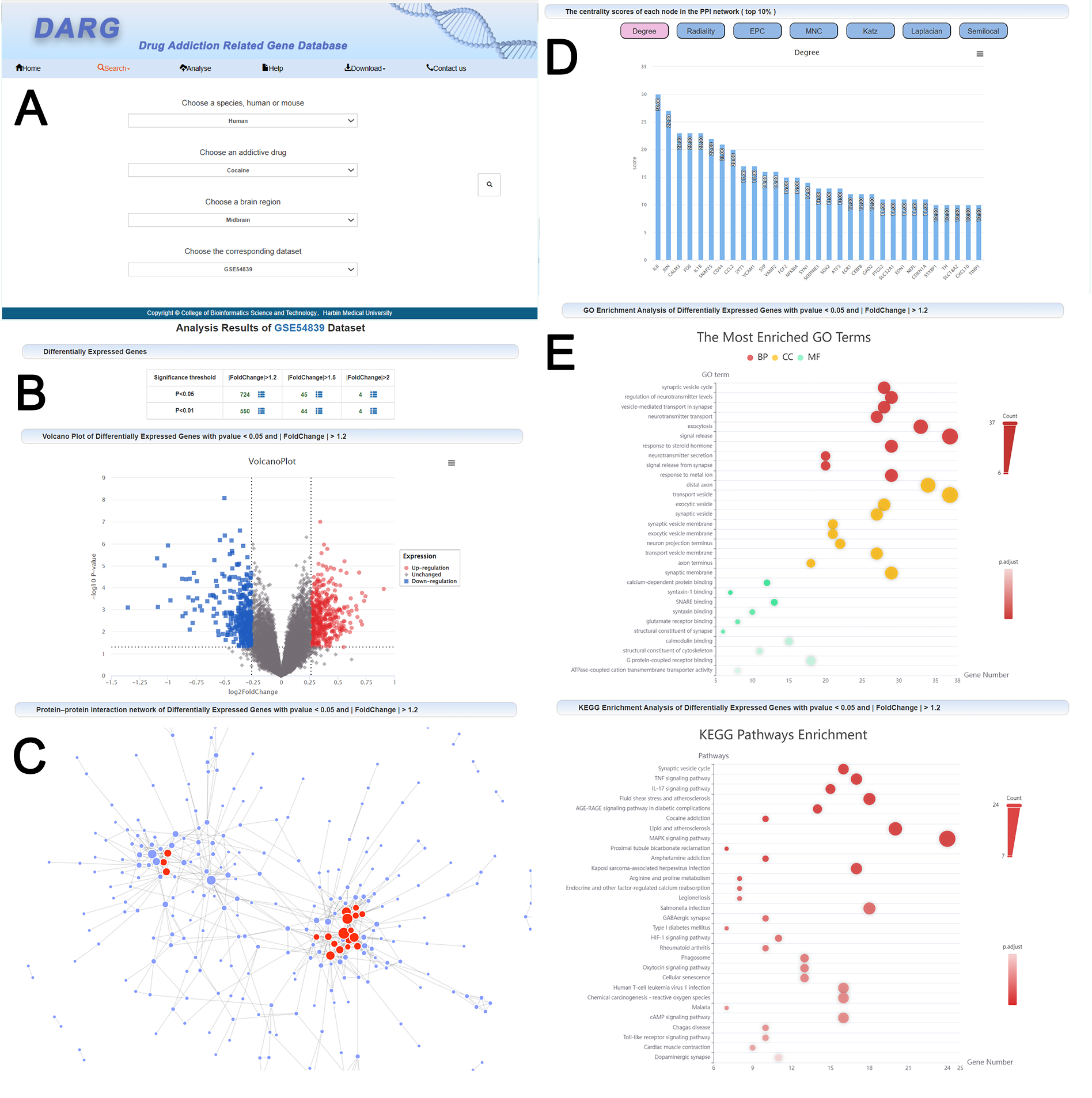


**Figure S1** Transcriptome data Search page and analyse results: (A) Transcriptome data Search page. (B) DEGs table with different thresholds and volcano plots of DEGs. (C) Differential gene interaction network. (D) Centrality algorithm results. (E) GO enrichment result and KEGG pathway result.


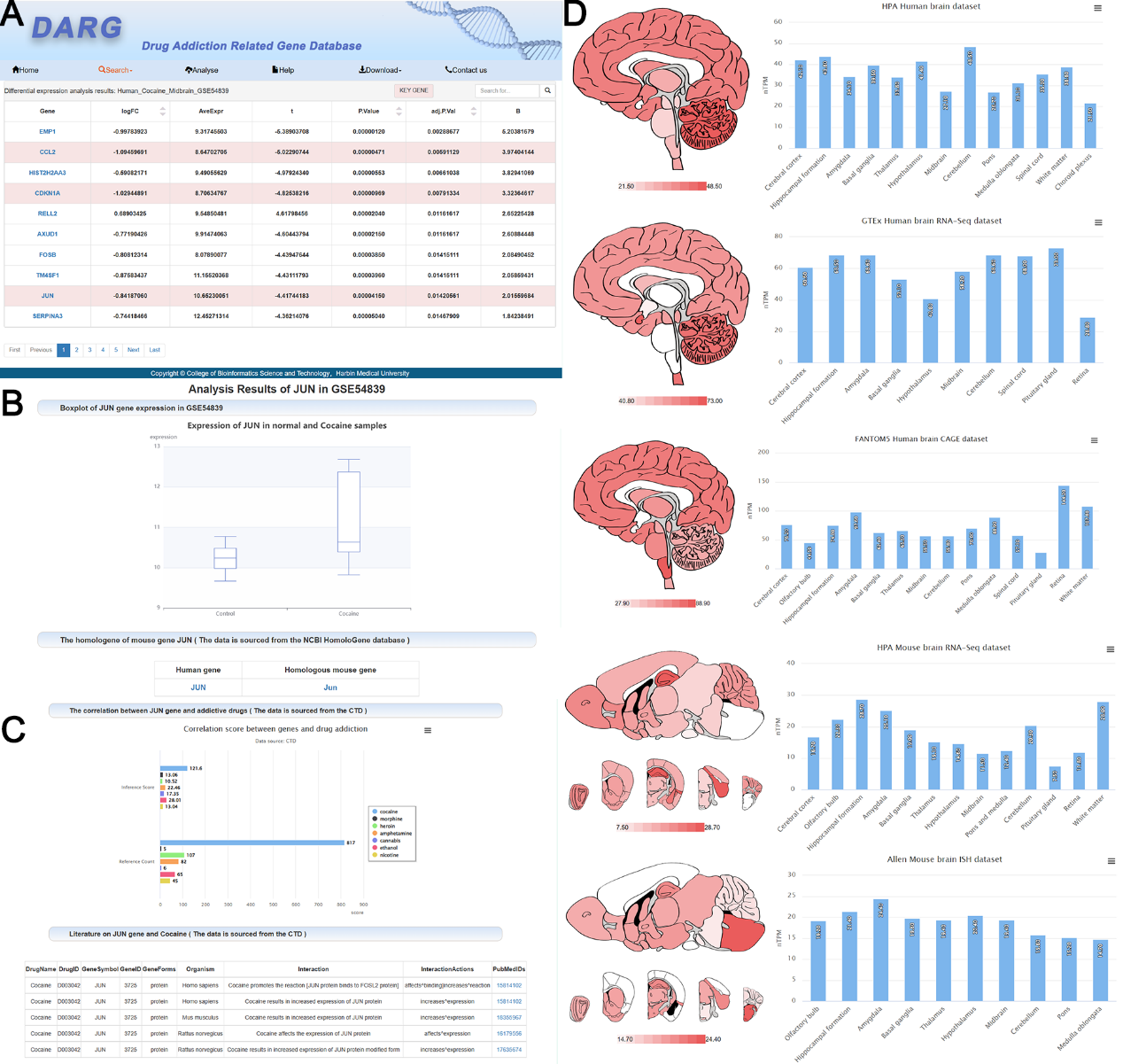


**Figure S2** DEGs list and detailed information: (A) Detailed list of DEGs. (B) Box plot of gene expression levels and homologous gene information. (C) Correlation between gene and addictive drugs and literature information on gene and addictive drugs. (D) RNA expression in human and mouse brain regions.


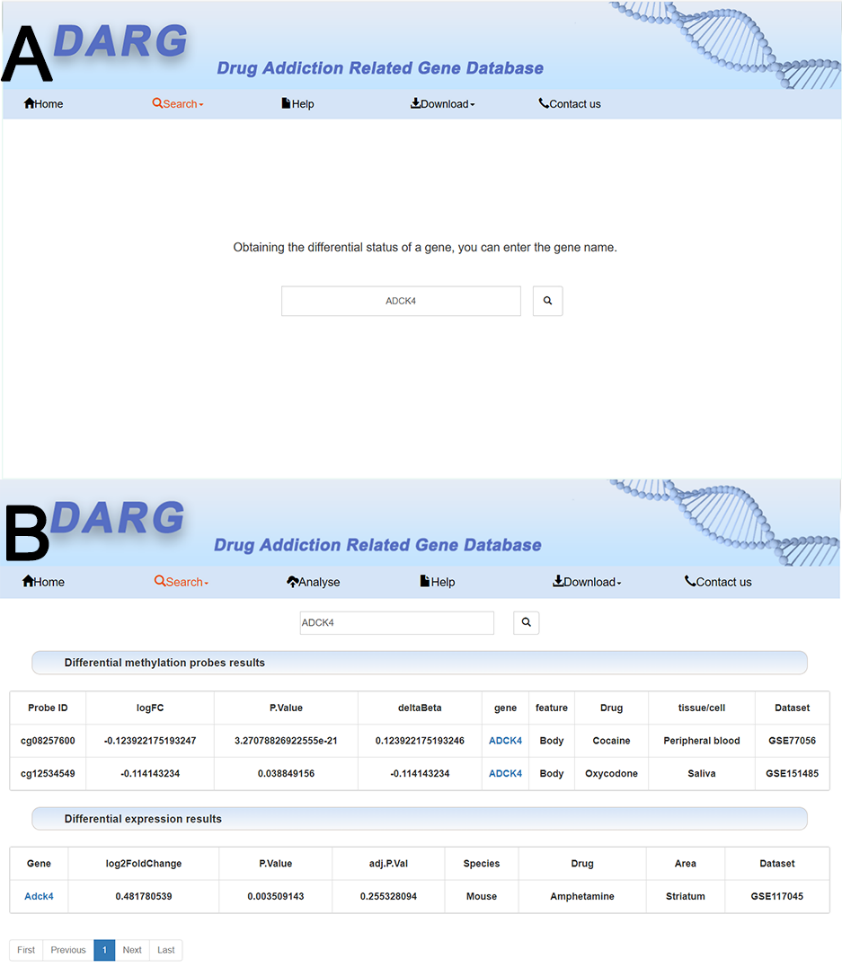


**Figure S3** Gene Search page and result: (A) Gene Search page. (B) Search results for differential expression and differential methylation.


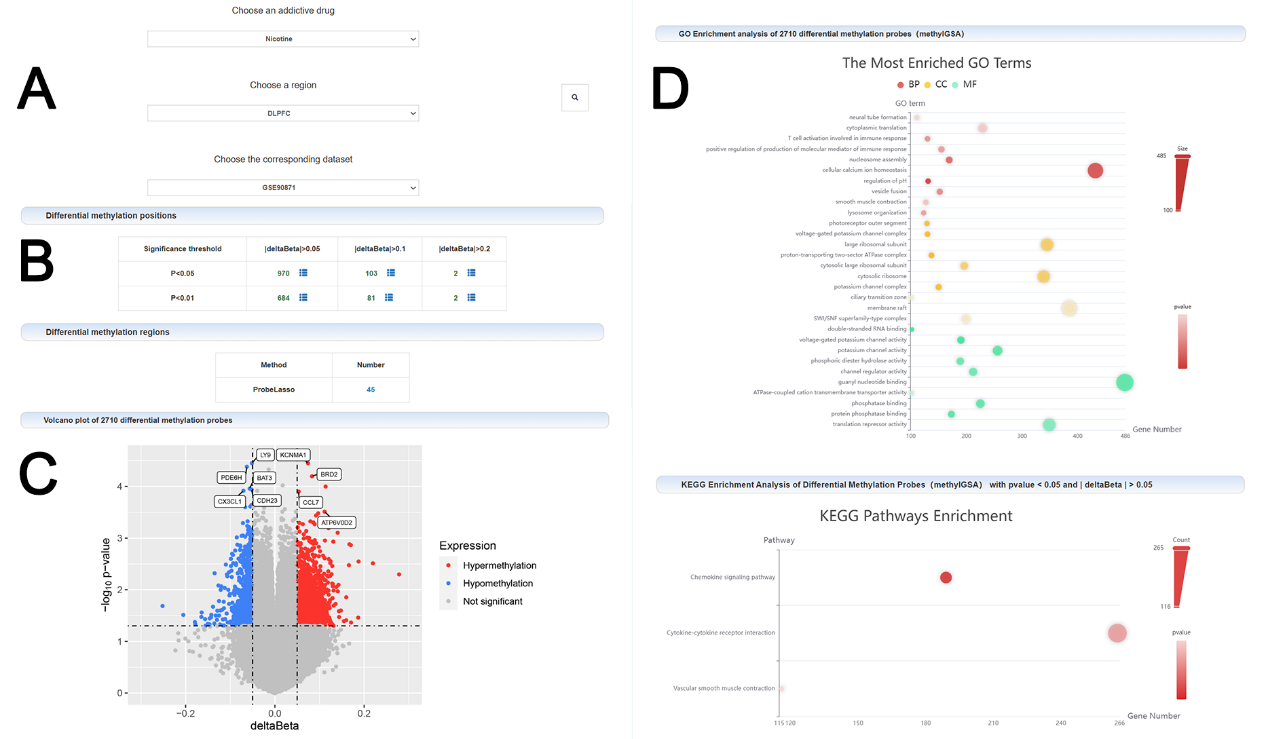


**Figure S4** Methylation data Search page and detailed information: (A) Methylation data Search page. (B) DMPs table with different thresholds and DMRs table. (C) Volcano map of DMPs. (D) GO enrichment result and KEGG pathway result.


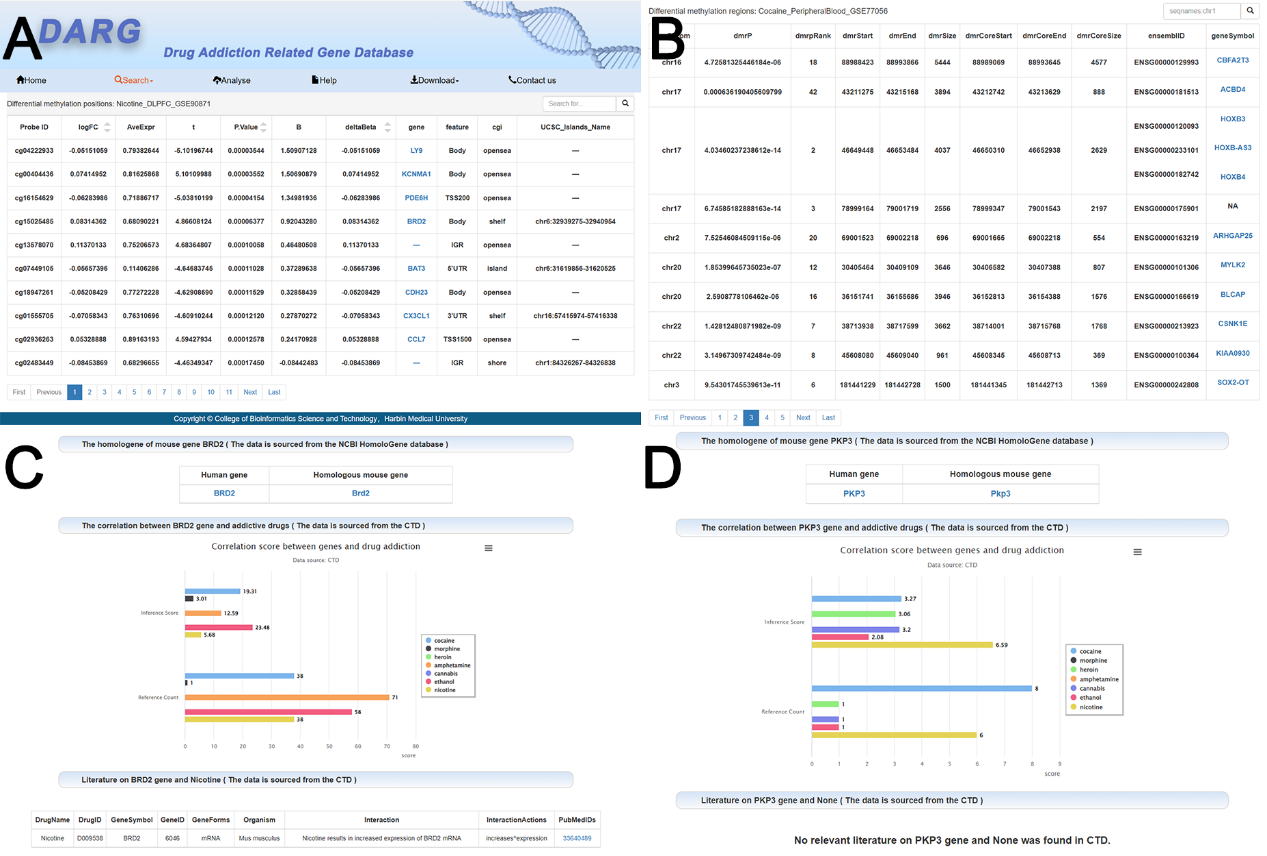


**Figure S5** DMPs List and Gene Details: (A) Detailed list of DMPs and related genes. (B) Detailed list of DMRs and related genes. (C) Detailed analysis results of genes corresponding to DMPs. (D) Detailed analysis results of genes corresponding to DMRs.


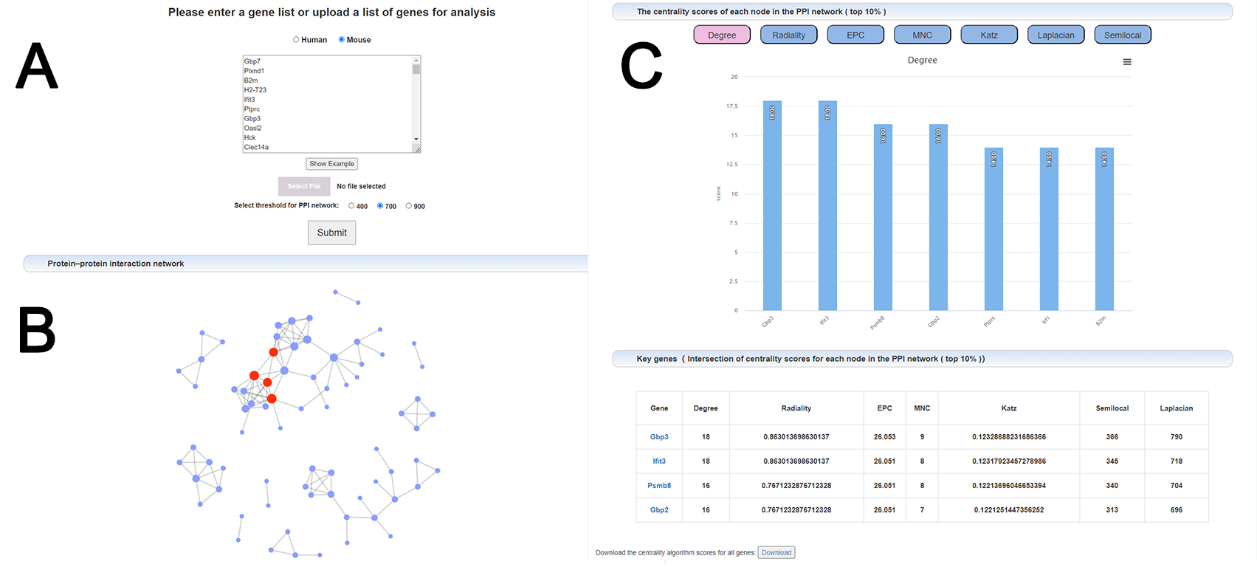


**Figure S6** Analyse upload and result page: (A) Analyse upload page. (B) Gene interaction network. (C) Centrality algorithm results and key gene list.


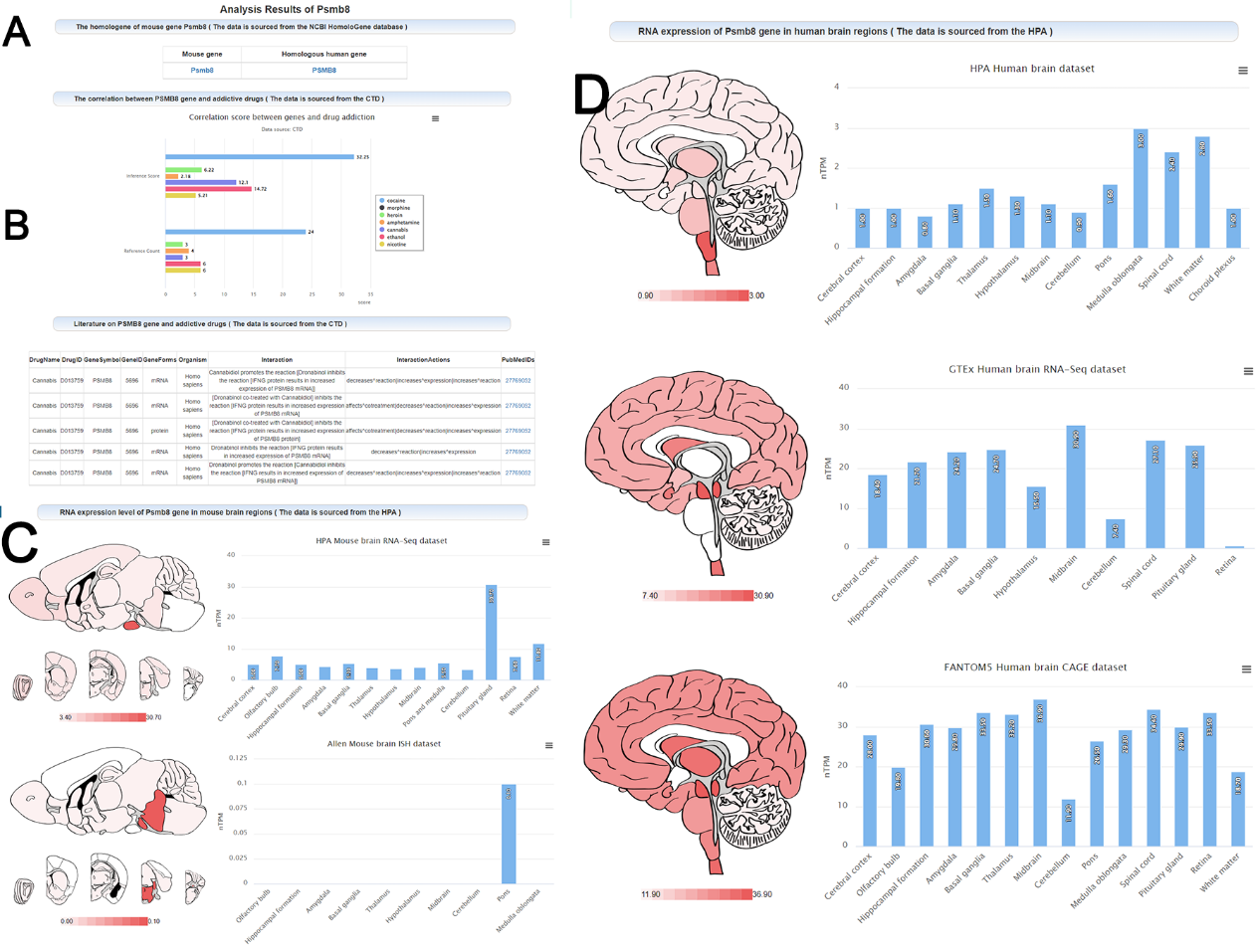


**Figure S7** Key gene page in the analysis results: (A) Homologous gene information. (B) Correlation between gene and addictive drugs and literature information on gene and addictive drugs. (C) RNA expression in mouse brain regions. (D) RNA expression in human brain regions.


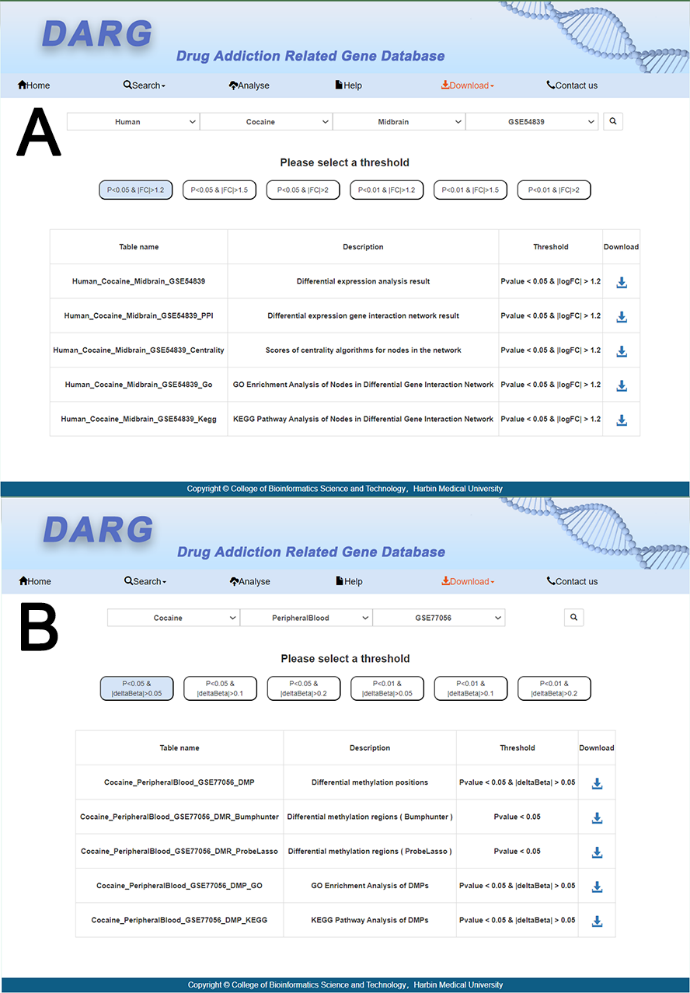


**Figure S8** Download page: (A) Transcriptome data analyse results download page. (B) Methylation data analyse results download page.
